# Rare Germline Variants in Chordoma-Related Genes and Chordoma Susceptibility

**DOI:** 10.3390/cancers13112704

**Published:** 2021-05-30

**Authors:** Sally Yepes, Nirav N. Shah, Jiwei Bai, Hela Koka, Chuzhong Li, Songbai Gui, Mary Lou McMaster, Yanzi Xiao, Kristine Jones, Mingyi Wang, Aurelie Vogt, Bin Zhu, Bin Zhu, Amy Hutchinson, Meredith Yeager, Belynda Hicks, Brian Carter, Neal D. Freedman, Laura Beane-Freeman, Stephen J. Chanock, Yazhuo Zhang, Dilys M. Parry, Xiaohong R. Yang, Alisa M. Goldstein

**Affiliations:** 1 Division of Cancer Epidemiology and Genetics, National Cancer Institute, National Institutes of Health, Bethesda, MD 20892, USA; nirav.shah2@nih.gov (N.N.S.); hela.koka@nih.gov (H.K.); Mary.McMaster@nih.gov (M.L.M.); yanzi.xiao@nih.gov (Y.X.); kristine.jones@nih.gov (K.J.); mingyi.wang@nih.gov (M.W.); vogta@mail.nih.gov (A.V.); bin.zhu@nih.gov (B.Z.); bin.zhu2@nih.gov (B.Z.); hutchiam@mail.nih.gov (A.H.); yeagerm@mail.nih.gov (M.Y.); hicksbel@mail.nih.gov (B.H.); freedmanne@mail.nih.gov (N.D.F.); freemala@mail.nih.gov (L.B.-F.); chanocks@mail.nih.gov (S.J.C.); parryd@mail.nih.gov (D.M.P.); royang@mail.nih.gov (X.R.Y.); 2 Beijing Tiantan Hospital, Beijing 100070, China; baijiwei@ccmu.edu.cn (J.B.); lichuzhong@ccmu.edu.cn (C.L.); guisongbai@ccmu.edu.cn (S.G.); zhangyazhuo@ccmu.edu.cn (Y.Z.); 3 Cancer Genomics Research Laboratory, Leidos Biomedical Research, Frederick National Laboratory for Cancer Research, Frederick, MD 21702-1201, USA; 4 American Cancer Society, Inc, Atlanta, GA 30303, USA; brian.carter@cancer.org

**Keywords:** chordoma, germline variants, notochord development, cancer susceptibility, WES, WGS

## Abstract

**Simple Summary:**

Chordoma is an extremely rare bone cancer that has not been fully characterized and few risk factors have been identified, highlighting the need for improving our understanding of the disease biology. Our study aims to identify chordoma susceptibility genes by investigating 265 genes involved in chordoma-related signaling pathways and other biological processes on germline DNA of 138 chordoma patients of European ancestry compared to internal control datasets and general population databases. Results were intersected with whole genome sequencing data from 80 skull-base chordoma patients of Chinese ancestry. Several rare loss-of-function and predicted deleterious missense variants were enriched in chordoma cases in both datasets, suggesting a complex model of pathways potentially involved in chordoma development and susceptibility, warranting further investigation in larger studies.

**Abstract:**

Background: Chordoma is a rare bone cancer with an unknown etiology. TBXT is the only chordoma susceptibility gene identified to date; germline single nucleotide variants and copy number variants in TBXT have been associated with chordoma susceptibility in familial and sporadic chordoma. However, the genetic susceptibility of chordoma remains largely unknown. In this study, we investigated rare germline genetic variants in genes involved in TBXT/chordoma-related signaling pathways and other biological processes in chordoma patients from North America and China. Methods: We identified variants that were very rare in general population and internal control datasets and showed evidence for pathogenicity in 265 genes in a whole exome sequencing (WES) dataset of 138 chordoma patients of European ancestry and in a whole genome sequencing (WGS) dataset of 80 Chinese patients with skull base chordoma. Results: Rare and likely pathogenic variants were identified in 32 of 138 European ancestry patients (23%), including genes that are part of notochord development, PI3K/AKT/mTOR, Sonic Hedgehog, SWI/SNF complex and mesoderm development pathways. Rare pathogenic variants in COL2A1, EXT1, PDK1, LRP2, TBXT and TSC2, among others, were also observed in Chinese patients. Conclusion: We identified several rare loss-of-function and predicted deleterious missense variants in germline DNA from patients with chordoma, which may influence chordoma predisposition and reflect a complex susceptibility, warranting further investigation in large studies.

## 1. Introduction

Chordoma is a rare, malignant bone tumor that occurs in the axial skeleton at cranial, spinal and sacral sites. Chordoma tumors are considered slow growing; however, local recurrences are frequent and treatment options are limited, particularly for those with advanced disease, highlighting the need for improving our knowledge of the disease biology and the discovery of novel druggable targets [1,2,3]. The clinical progression of skull base chordoma is highly variable [4] and there are no validated clinical or molecular prognostic markers.

Chordoma is hypothesized to originate from remnants of the notochord, which is a mesodermal structure in the embryo and signal tissues for organization and differentiation. Genes implicated in chordoma formation include the T-brachyury gene (*TBXT*), which is required for differentiation of the notochord and formation of mesoderm during posterior development [5].

*TBXT* is the most relevant susceptibility gene identified in chordoma, with germline *TBXT* duplication conferring susceptibility to familial chordoma [6] (Yang XR et al. 2009) and single-nucleotide polymorphisms (SNPs) in *TBXT* associated with chordoma risk in both sporadic and familial chordoma [7,8]. The role of *TBXT* in disease pathogenesis has also been shown to be critical: *TBXT* expression is considered as a diagnostic marker for chordoma [9,10] and copy number gains of *TBXT* have been identified in chordoma tumors [11,12]. Further, chordoma growth in cell models could be inhibited by *TBXT* silencing [12,13,14].

Despite some progress, etiologic factors and genetic susceptibility of chordoma are not well understood and have not been extensively studied. The goal of this study was to identify and characterize rare and pathogenic germline variants in genes of interest for chordoma pathogenesis through next generation sequencing of 138 chordoma cases of European ancestry. Genes of interest from the initial evaluation were also examined in a set of 80 skull-base chordoma cases from an Asian population for replication. Due to the critical relevance of *TBXT* in the biological mechanisms of chordoma, we investigated germline variants in genes related to *TBXT*, as well as other genes involved in mesoderm and notochord development. We also evaluated genes that have been associated with chordoma tumor development, including several members of the SWI/SNF complex, PI3K/AKT/mTOR and Sonic Hedgehog pathways. Using this approach, we identified several potential chordoma susceptibility genes that warrant investigation in future studies.

## 2. Materials and Methods

### 2.1. Study Populations

The current study included 138 patients of European ancestry from the United States and Canada, processed using whole exome sequencing (WES). All diagnoses of chordoma were confirmed by reviewing pathologic slides or reports, medical records or death certificates.

We used WES data from 598 healthy Caucasian cancer-free unrelated individuals, from the Prostate, Lung, Colorectal and Ovarian Cancer Screening Trial (PLCO) [15] and Cancer Prevention Study (CPS) as reference/controls for comparison with the European ancestry patient samples and for conducting rare variant burden tests. These controls were sequenced and analyzed using the same sequencing platform and ensemble variant calling pipeline used for the European ancestry chordoma cases.

We also analyzed germline whole genome sequencing (WGS) data from 80 Chinese skull-base chordoma patients to evaluate candidate genes identified from our main analysis of the European ancestry chordoma patients. In the Chinese patient cohort, all patients were diagnosed with skull base chordoma and underwent endoscopic endonasal surgeries at the Neurosurgery Department of Beijing Tiantan Hospital, Capital Medical University, between October 2010 and November 2017. All methods were performed in accordance with the relevant guidelines and regulations. This research has been approved by the National Cancer Institute (NCI) Institutional Review Board (IRB) (Protocols: 10CN188, 04/08/2020; 78C-0039, 01/08/2021) and the ethics committee of the Beijing Tiantan Hospital (IRB code: 2009-47, 20 December 2009).

### 2.2. Library Construction, Sequencing and Bioinformatics Analysis

For the European ancestry and Chinese cases, germline genomic DNA was extracted from saliva and peripheral blood. WES was performed at the Cancer Genomics Research Laboratory, National Cancer Institute (CGR, NCI). Briefly, SeqCAP EZ Human Exome Library v3.0 (Roche NimbleGen, Madison, WI, USA) was utilized for exome sequence capture. Exome sequencing was performed to a sufficient depth to achieve a minimum coverage of 15 reads in at least 80% of the coding sequence from the UCSC hg19 transcripts database.

After the exclusion of reads containing adapter contamination and low-quality nucleotides, the data were mapped to the reference human genome (UCSC hg19) using the Burrows–Wheeler Aligner software [16]. SAMtools [17], Picard and GATK [18] were used to sort aligned files and perform base quality recalibration, duplicate reads removal and local realignment to generate final BAM files for mutation calling.

Three variant callers, GATK HaplotyperCaller, UnifiedGenotyper and FreeBayes [19], were used to call germline variants. We included all target regions, as well as a 250 bp flanking region on each side. An ensemble variant calling pipeline was then implemented to integrate the analysis results from the above mentioned three callers. Subsequently, the ensemble variant calling pipeline that applies a support-vector machine (SVM) learning algorithm was used to identify an optimal decision boundary based on the variant calling results out of the multiple variant callers to produce a more balanced decision between false positives and true positives.

For the Chinese cases, WGS was carried out by the Novogene Corporation (Beijing, China) on the Illumina HiSeq X platform with an average depth of 41X. Variant calling, QC and filtering steps were conducted by CGR, NCI, using similar approaches as those used for WES of the European ancestry chordoma samples.

**Gene prioritization:** We assembled a set of 265 genes chosen from the published literature for their role in chordoma physiopathology. The following functional categories were included: *TBXT* super-enhancer associated genes and chordoma essentiality genes, which are essential for the proliferation and survival of cancer cells in CRISPR-CAS9 screens, notochord related genes, key genes in mesoderm commitment pathway, SWI/SNF complex, PI3K/Akt/mTOR pathway and Sonic Hedgehog pathway. Variants were filtered based on quality control measures, frequency of minor allele in populations and pathogenicity criteria.

**Variants quality control:** Filtering of WES variants was based on the following criteria: Variants flagged with our pipeline quality control metric (CScorefilter), read depth <10, ABHet <0.2 or >0.8, or called by only one of the three variant callers used were excluded. Only non-synonymous variants, including frameshift, stop/gain, inframe deletion or insertion, missense and splicing site variants, were studied.

**Frequency and specificity:** Variants were excluded if both of the following criteria were met: (1) they had a minor allele frequency (MAF) of >0.001 in any of the consulted population databases (the 1000 Genomes Project, Exome Sequencing Project (ESP6500) and Exome Aggregation Consortium (ExAC) in European population); (2) they were observed in >2 families from an in-house database (CGR, NCI) of ~2000 exomes in ~1000 cancer-prone families, excluding chordoma cancer families. 

**Pathogenicity evidence and in silico analysis:** Variants passing quality control and frequency filters in both datasets were evaluated for pathogenicity. Variants were studied further if classified as one of the following: (1) High impact variants (frameshift indels, stop gain/loss, or known splice sites). (2) Missense variants with evidence of pathogenicity based on 3 in silico predictions (Meta Likelihood ratio: D, METASVM: D and CADD: ≥ 20). The first two in silico algorithms are ensemble prediction scores that incorporate results from nine algorithms (SIFT, PolyPhen-2, GERP ++, Mutation Taster, Mutation Assessor, FATHMM, LRT, SiPhy and PhyloP) and allele frequency [20]. (3) Variants classified as pathogenic (P) or likely pathogenic (LP) by ClinVar [21]. (4) Disease-causing mutation (DM) by HGMD [22]. Details of the criteria for classification of pathogenicity are shown in Supplementary Table S1 [23].

Similar filtering as described for the European ancestry dataset was performed in the Chinese dataset processed with WGS. For this latter dataset, only protein-coding genes, for which potentially pathogenic variants were observed in the European ancestry dataset, were considered. Variants were excluded based on MAF of >0.001 in any of the databases in any population, including East Asian. Pathogenicity evidence and in silico analysis were performed as described above for the European ancestry dataset.

### 2.3. Rare Variant Burden Test

We conducted rare variant burden tests for genes with rare and potentially pathogenic variants identified from the European ancestry cohort in 138 chordoma patients and 598 non-cancer control samples from the PLCO and CPS-II studies with European ancestry and processed at CGR, NCI. We used SKAT-O statistics [24], which is a linear combination of the burden test (aimed to test effect size of variants with the same direction in cases and controls) and variance component test (designed to test effect size of variants with different directions in cases and controls).

## 3. Results

The main analysis included 138 European ancestry patients; the average age at diagnosis was 46.9 years (range 7–78) and the ratio of male to female patients was 0.7. The vast majority of patients (97%) had classic chordoma histology. The chordoma site distribution was 55.7% skull base, 23% spinal and 20.3% sacral. The Chinese cases were all skull base chordoma, with a mean age of 44.7 (7–79) years, the majority being male (62.5%) and having classic chordoma histology (80%).

**Figure 1:**
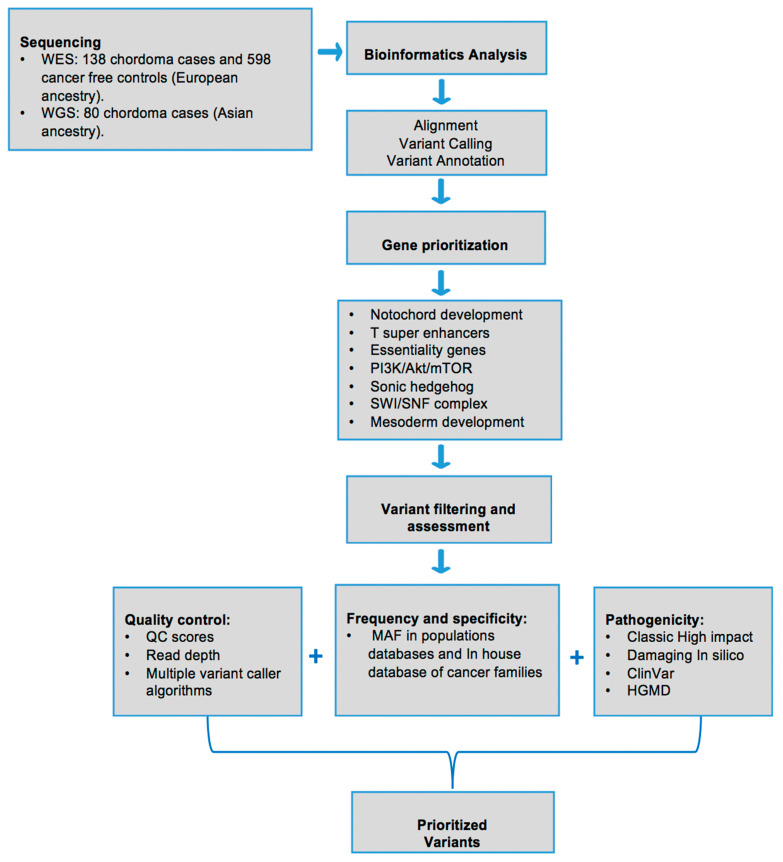
Overview of study samples and prioritization pipeline to evaluate rare and potentially pathogenic variants in genes of interest for chordoma.

We assembled a set of 265 genes chosen from the published literature for their role in chordoma physiopathology, including *TBXT* super-enhancer associated genes and chordoma essentiality genes in CRISPR-CAS9 screens (26), notochord related genes (21), mesoderm commitment pathway (166), SWI/SNF complex (18), PI3K/Akt/mTOR pathway (33) and Sonic Hedgehog pathway (28). Some of these genes are present in more than one category (Supplementary Table S2). Figure 1 shows the stepwise pipeline used for selecting, prioritizing and filtering the genes and variants for the evaluation.

We identified 34 potentially pathogenic variants (7 loss of function and 27 missense variants) in 2 notochord development genes, 4 Sonic Hedgehog pathway genes, 16 mesoderm commitment pathway genes, 4 PI3K/AKT/mTOR pathway genes, 2 SWI/SNF complex pathway genes, 2 *TBXT* super-enhancer genes and 1 driver gene (Table 1). These variants were identified in 32 of 138 European ancestry patients (23%). Table 1 and Table 2 show patients’ characteristics, the type, location and frequencies of these variants, evidence for pathogenicity and the involved pathways/biologic processes. Supplementary Tables S3 and S4 show details of the variants identified in the European ancestry and Chinese datasets, respectively.

Several variants were classified as DM/P by HGMD/ClinVar, such as *GDF3* (rs146973734), *AKT1* (rs397514644) and *EXT2* (rs138495222). Many of the variants were either extremely rare in the general population or absent from the internal control samples and population databases such as variants identified in *TBXT*, *ATP6V1B2* and *COL2A1*.

**Burden test:** To identify genes with a higher genetic burden of rare variants, we conducted a rare variant burden test of the 265 genes by comparing cases to controls of the same ancestry and sequenced using similar analytics and pipelines. Burden tests were performed in several different ways: (1) analyzing all rare variants in a targeted gene; (2) examining only rare pathogenic variants in a targeted gene; (3) examining all genes in a process or pathway. The small number of cases carrying variants in the same genes limited the power to evaluate statistical significance for individual genes. As a result, none of the examined genes showed statistical significance after correction for multiple testing (Supplementary Table S5). However, fourteen rare pathogenic variants were found only in chordoma cases and were absent from all 598 controls (variants in these genes: *AKT1, ATP6V1B2, DEPTOR, FOXA2, HHIP, NFE2L2, PAX6, PDK1, PRKACA, PTCH1, SMARCB1, SOX21, SOX9* and *TBXT*). Evaluation of the biologic processes or pathways showed suggestive evidence for association only for the notochord-related genes (SKATO P = 0.06; Burden P = 0.03).

We investigated the 31 genes (see Table 1) identified in the main analysis in an independent dataset of 80 Chinese chordoma cases. After applying the same filtering criteria as for the main analysis (see methods), we identified rare pathogenic variants in 11 genes in 18 of 80 (22.5%) Chinese patients. Most variants were very rare, with MAFs ranging between 1.74 E–04 and zero in all control databases (Table 2). Although most genes/variants were observed in only a single Chinese chordoma case, multiple potentially pathogenic variants were observed in *COL2A1, LRP2, TCF7L1* and *TSC2*. In particular, the two variants in *TSC2* were observed in two (g. 2134572) or three (g. 2121610) different patients, respectively. Further, the *TSC2* variant observed in three Chinese chordoma cases was classified as DM by HGMD (Table 2).

## 4. Discussion

In this study, we investigated the role of rare germline variants in genes involved in chordoma related processes/pathways in sporadic chordoma patients from a European ancestry and a Chinese population. We identified a number of rare loss-of-function and predicted deleterious missense variants that were enriched in chordoma cases, suggesting some of these genes may contribute to chordoma susceptibility.

Genes with rare germline variants enriched in chordoma cases were involved in signaling pathways and physiopathological processes that are affected in chordoma at the somatic level (e.g., notochord development, PI3K/AKT/mTOR and SWI/SNF pathways), which underscores the importance of pathway/process-level analysis of germline alterations to identify potentially novel genes. Increasing evidence suggests that specific germline variants might determine which somatic events and mutations are generated and selected in cancer cells during tumorigenesis, such as mutational signatures, allele-specific copy number changes, or altered signaling pathways [25].

Overall, we report that ~23% of chordoma cases from two independent populations carried rare germline variants that are potentially pathogenic in selected genes. Eleven genes were shared by the two datasets, including COL2A1 (notochord), EXT1 (mesoderm development), PDK1 (PI3K/Akt/mTOR) and LRP2 (Sonic Hedgehog) and thus worthy of additional studies.

Some of the genes identified in this study have previously demonstrated important biological functions relevant to chordoma development. For example, a recent study found that *TBXT* is associated with a 1.5 Mb region containing “super-enhancers” and is the most highly expressed super-enhancer-associated transcription factor [26]. *ATP6V1B2* was among the genes that were considered as both super-enhancer-associated and essential for chordoma cell viability. sgRNA-mediated *TBXT* repression experiments have been shown to reduce the expression of this gene and the direct binding of *TBXT* at the *ATP6V1B2* locus has been demonstrated [26]. *COL2A1* was also shown to be down-regulated by *TBXT* expression in a clival chordoma cell line (UM-Chor1) [26]. Interestingly, Col2a1-null mice lack intervertebral discs and cannot remove the notochord [27], suggesting the potential importance of this gene in chordoma development. We also identified rare variants in *SHH*, a member of the Sonic Hedgehog pathway, which is secreted by fetal notochord and participates in vertebrate patterning of the neural tube during embryonic development via binding to its ligand *PTCH1*. Interestingly, Shh-expressing notochord cells remain in the vertebral column after embryonic development ends, resembling notochord remnants [28] and suggesting that it may cause transformation of this remnant tissue.

The *SMARCB1* gene is a critical component of the SWI/SNF chromatin-remodeling complex which antagonizes the histone methyltransferase EZH2, a molecule that is being targeted by several inhibitors in clinical trials [29]. Inactivation of *SMARCB1* has been described as a critical event in various tumors, including malignant rhabdoid tumors and epithelioid sarcoma [30]. Loss of *SMARCB1* expression in chordoma has also been identified in poorly differentiated chordomas [31]. Recently, other components of SWI/SNF complex have also been implicated in chordoma pathogenesis, such as *PBRM1* as a potential driver gene for chordoma [11].

PI3K/Akt/mTOR signaling in the context of chordoma was first suggested after numerous reports of chordoma tumors in individuals with tuberous sclerosis complex (TSC). Chordoma also occurs in association with TSC, an autosomal dominant neurocutaneous syndrome characterized by abnormal tissue growth in multiple organ systems caused by inactivating germline mutations in either *TSC1* or *TSC2* [32]. None of the patients in our study with *TSC* variants have clinical manifestations of TSC. Nevertheless, mutations in PI3K/AKT/mTOR genes have been reported in chordoma tumors with potential therapeutic relevance [11].

The strengths of our study include the careful and comprehensive literature review to select chordoma candidate genes, the evaluation of germline rare variants in two independent chordoma sequencing datasets and the inclusion of controls without cancer from the same population ancestry as our European ancestry chordoma cases that were processed using the same platforms and bioinformatics pipeline. However, we cannot rule out the possibility that other genes, including cancer-predisposing genes, may also play a role in susceptibility.

The main limitation of our study is the relatively small number of chordoma cases in each dataset to statistically test case-control differences at an individual gene level, with most variants found only in single chordoma cases. In addition, we were unable to assess family history for most cases. There was also limited representation of histological subtypes for statistical evaluation of association with the variants identified.

In this study, approximately 23% of patients with chordoma from two different population had rare pathogenic/likely pathogenic variants in various biological processes, suggesting a complex model of pathways potentially important for susceptibility and development, including notochord development, PI3K/AKT/mTOR, Sonic Hedgehog and SWI/SNF complex. Future large studies, particularly at the consortium level, are needed to follow up on our findings for a better understanding of genetic susceptibility of this extremely rare cancer.

## 5. Conclusions

Our study searched for germline variants in signaling pathways and other biological processes previously identified in the disease’s pathogenesis in patients from two independent populations. We identified rare loss-of-function and predicted deleterious missense variants enriched in chordoma cases, suggesting a complex model of pathways potentially involved in chordoma development and susceptibility. Further evaluation of the identified candidate genes is needed to determine their importance in chordoma risk.

## Figures and Tables

**Table 1:** Information on rare variants identified in the European ancestry patients after the prioritization procedure.

ID	Gender	Age	Morphology	Site	Gene	Chr	Location	SNP IDS	REF	VAR	Variant Type	Protein Change	Variant Impact	Pathogenecity Prediction ^a^			HGMD/ ClinVar	MAF in Control Datasets		Pathway/Process
														METASVM	METALR	CADD		gnomAD Exomes NFE ^b^	gnomAD Genomes NFE ^c^	
1004	Female	NA	Conventional	1	ATP8B2	1	154305114		G	T	missense	Arg210Leu	moderate	D	D	34		1.06 × 10^−5^		Mesoderm commitment
1101	Male	37.2	Conventional	2	LYST	1	235922291		G	A	stop_gained	Arg2288 *	high							Driver
1164	Male	29.7	Conventional	1	TCF7L1	2	85529694	rs147750102	G	A	missense	Gly205Ser	moderate	D	D	22.4		9.67 × 10^−5^	6.49 × 10^−5^	Mesoderm commitment
1040	Female	32.7	Conventional	1	EPB41L5	2	120776677	rs200315720	G	A	missense	Arg6His	moderate	D	D	29.9		1.98 × 10^−4^	1.30 × 10^−4^	Mesoderm commitment
1020	Female	39.0	Conventional	1	EPB41L5	2	120833086	rs766560121	C	A	missense	Leu148Ile	moderate	D	D	28.9		0		Mesoderm commitment
3001	NA	NA	NA	NA	LRP2	2	170038097	rs137983840	C	T	missense	Ala3344Thr	moderate	D	D	26.3		6.16 × 10^−5^	6.48 × 10^−5^	Sonic Hedgehog
1035	Female	26.1	Chondroid	1	PDK1	2	173460594			T	frameshift & stop_gained	Asn424fs	high							PI3K/AKT/mTOR
1111	Male	51.3	Chondroid	1	NFE2L2	2	178095743		C	A	missense	Asp530Tyr	moderate	D	D	22.5				Mesoderm commitment
1014	Female	44.3	Conventional	1	BMPR2	2	203420750	rs146310981	G	A	missense	Val788Ile	moderate	D	D	22.7		3.52 × 10^−5^		Mesoderm commitment
1139	Female	39.5	Conventional	1	RARB	3	25611341		A	G	missense	Thr188Ala	moderate	D	D	22.9		8.79 × 10^−6^		Mesoderm commitment
1053	Female	54.6	Conventional	1	HHIP	4	145581068			ACAC	frameshift	Phe304fs	high					0		Sonic Hedgehog
1127	Male	50.7	Conventional	3	SRF	6	43146888	rs765592889	T	C	missense	Val496Ala	moderate	D	D	22.4		1.76 × 10^−5^		Mesoderm commitment
1048/ 5320	NA	NA	NA	NA	TBXT	6	166571981	rs368179445	C	T	missense	Arg377Gln	moderate	D	D	25.5		0		T super-enhancer
1080	Female	47.4	Conventional	1	ATP6V1B2	8	20068082		C	T	missense & splice region	Arg130Trp	moderate	D	D	35		0		T super-enhancer
1161	Male	78.3	Conventional	1	EXT1	8	118832021	rs145720047	G	C	missense	Pro477Arg	moderate	D	D	22.4		1.24 × 10^−4^	6.48 × 10^−5^	Mesoderm commitment
1001	Female	47.0	Conventional	1	DEPTOR	8	120977595			A	frameshift	Glu185fs	high							PI3K/AKT/mTOR
1034	Female	51.0	Conventional	2	SMARCA2	9	2039568	rs774084308	C	T	missense	Pro153Leu	moderate	D	D	22.2		0		SWI/SNF complex
1080	Female	47.4	Conventional	1	JAK2	9	5078325		A	G	missense	His671Arg	moderate	D	D	21.7				Mesoderm commitment
1154	Female	44.1	Conventional	2	PTCH1	9	98209505	rs556901417	G	A	missense	Arg1345Cys	moderate	D	D	25.2		1.09 × 10^−4^	3.89 × 10^−4^	Sonic Hedgehog
1042	Female	39.5	Conventional	1	SUFU	10	104309821	rs34406289	G	A	missense	Ala138Thr	moderate	D	D	33		2.64 × 10^−5^	0	Sonic Hedgehog
1141	Female	57.6	Conventional	3	SUFU	10	104375030	rs79299301	G	A	missense	Arg343His	moderate	D	D	20.8		1.58 × 10^−4^	6.49 × 10^−5^	Sonic Hedgehog
1006	Male	46.2	Conventional	1	PAX6	11	31823215		A	T	missense	Val98Glu	moderate	D	D	27.5				Mesoderm commitment
1162	Female	66.4	Conventional	3	EXT2	11	44254000	rs138495222	C	T	missense	Thr620Met	moderate	D	D	34	DM	9.24 × 10^−4^	8.43 × 10^−4^	Mesoderm commitment
1090	Female	28.3	Conventional	1	GDF3	12	7842985	rs146973734	C	T	missense	Arg195Gln	moderate	T	T	0.004	P/DM	1.85 × 10^−4^	5.84 × 10^−4^	Notochord development
1001	Female	47.0	Conventional	1	COL2A1	12	48380213	rs201823490	G	A	missense	Pro478Leu	moderate	D	D	23.7		0	0	Notochord development
1113	Female	27.8	Conventional	1	SOX21	13	95364265		G	C	missense	Asn13Lys	moderate	D	D	21.6				Mesoderm commitment
1050	Male	60.3	Conventional	2	WDHD1	14	55462357	rs556223202	G	A	stop_gained	Arg373 *	high					8.88 × 10^−6^		Mesoderm commitment
1127	Male	50.7	Conventional	3	AKT1	14	105246527	rs397514644	G	A	missense	Arg25Cys	moderate	D	T	29.6	P/DM			PI3K/AKT/mTOR
1091	Female	69.9	Conventional	2	TSC2	16	2130319		C	T	missense	Ala1184Val	moderate	D	D	24		3.56 × 10^−5^		PI3K/AKT/mTOR
1028	Female	39.7	Conventional	1	TSC2	16	2136297	rs373635516	C	T	missense	Pro1589Leu	moderate	D	D	21.9		0		PI3K/AKT/mTOR
1006	Male	46.2	Conventional	1	ACACA	17	35603791		A	T	missense	Val804Glu	moderate	D	D	23.7				Mesoderm commitment
1114	Male	44.4	Conventional	1	PRKACA	19	14203391	rs41296324	A	T	stop_lost	Ter125Lysext *	high					0	2.60 × 10^−4^	Mesoderm commitment
1045	Female	31.2	Conventional	1	FOXA2	20	22563180		C	T	missense	Gly234Ser	moderate	D	D	22.3				Mesoderm commitment
1098	Female	13.8	Conventional	3	SMARCB1	22	24129394		AA		frameshift	Lys13fs	high							SWI/SNF complex

Site: 1, bones of skull and face and associate joints; 2, pelvic bones, sacrum and coccyx and associated joints; 3, vertebral column. Chr, chromosome; REF, reference allele; VAR, variant allele; Freq, frequency; MAF, minor allele frequency; T, tolerant; D, deleterious; P, pathogenic; DM, disease-causing mutation. ^a^ Pathogenicity prediction for missense variants based on in silico algorithms, METALR and METASVM, which are ensemble prediction scores that incorporate results from nine algorithms and allele frequency. CADD was also applied. ^b,c^ Genome Aggregation Database (gnomAD) exomes and genomes, respectively, in non-Finnish European population (NFE).

**Table 2:** Potentially pathogenic variants in the Chinese dataset.

ID	Gender	Age	Morphology	Gene	Chr	Location	SNP IDS	REF	VAR	Variant Type	Protein Change	Variant Impact	Pathogenecity Prediction ^a^			HGMD/ ClinVar	MAF in Control Datasets		Pathway/Process
													METASVM	METALR	CADD		gnomAD Exomes EAS ^b^	gnomAD Genomes EAS ^c^	
1804	M	65	Conventional	ATP8B2	1	154317567		A	C	missense	Lys836Gln	moderate	D	D	26.2		1.74 × 10^−4^	0	Mesoderm commitment
1686	M	64	Conventional	LRP2	2	170033035		C	T	missense	Gly3486Glu	moderate	D	D	33		0	0	Sonic Hedgehog
1372	NA	NA	NA	LRP2	2	170062924	rs577943281	T	A	missense	Thr2436Ser	moderate	D	D	22.7		2.90 × 10^−4^	6.17 × 10^−4^	Sonic Hedgehog
1964	M	24	Chondroid	LRP2	2	170055366	rs755215116	A	C	missense	Ile2836Met	moderate	D	D	24.2		3.66 × 10^−3^	6.17 × 10^−4^	Sonic Hedgehog
1442	NA	NA	NA	PDK1	2	173460575	rs745678398	A	G	structural interaction		High					0	0	PI3K/AKT/mTOR
1886	F	62	Conventional	TCF7L1	2	85536476		C	T	missense	Thr553Ile	moderate	D	D	23		5.82 × 10^−5^	0	Mesoderm commitment
1790	M	65	Conventional	TCF7L1	2	85531113	rs373770977	G	A	missense	Val252Ile	moderate	D	D	23.4		1.25 × 10^−4^	0	Mesoderm commitment
1779	M	47	Conventional	TCF7L1	2	85536536	rs555810312	C	T	missense	Pro573Leu	moderate	D	D	23.2		4.19 × 10^−4^	0	Mesoderm commitment
2011	F	7	Conventional	TBXT	6	166581010	rs563349798	C	G	missense	Val24Leu	moderate	D	D	29.7		0	0	T super-enhancer
1686	M	64	Conventional	EXT1	8	118819501	rs753261171	G	A	missense	Thr613Met	moderate	D	D	33		0	0	Mesoderm commitment
776	M	54	Conventional	SUFU	10	104309738		C	T	structural interaction		High					5.80 × 10^−5^	0	Sonic Hedgehog
1921	M	57	Conventional	EXT2	11	44193231	rs767085143	A	G	missense	Asn448Ser	moderate	D	D	24.3		2.90 × 10^−4^	0	Mesoderm commitment
1372	NA	NA	NA	COL2A1	12	48369322	rs995646562	C	T	missense	Ala1222Thr	moderate	D	D	24.1		0	0	Notochord development
1909	M	26	Conventional	COL2A1	12	48372528		G	T	missense	Pro916His	moderate	D	D	25		0	0	Notochord development
1936	F	71	Conventional	TSC2	16	2134572	rs45517338	C	G	missense	Pro1450Arg	moderate	D	D	25.8		2.35 × 10^−4^	0	PI3K/AKT/mTOR
1417	M	59	Conventional	TSC2	16	2134572	rs45517338	C	G	missense	Pro1450Arg	moderate	D	D	25.8		2.35 × 10^−4^	0	PI3K/AKT/mTOR
1979	M	59	Chondroid	TSC2	16	2121610	rs45509392	G	A	missense	Asp647Asn	moderate	D	D	32	DM	5.80 × 10^−4^	0	PI3K/AKT/mTOR
1570	M	24	Chondroid	TSC2	16	2121610	rs45509392	G	A	missense	Asp647Asn	moderate	D	D	32	DM	5.80 × 10^−4^	0	PI3K/AKT/mTOR
462	F	62	Conventional	TSC2	16	2121610	rs45509392	G	A	missense	Asp647Asn	moderate	D	D	32	DM	5.80 × 10^−4^	0	PI3K/AKT/mTOR
1084	NA	NA	NA	ACACA	17	35549102	rs772483773	C	T	missense	Val1449Met	moderate	D	D	25.2		6.96 × 10^−4^	6.17 × 10^−4^	Mesoderm commitment

Chr, chromosome; REF, reference allele; VAR, variant allele; Freq, frequency; MAF, minor allele frequency; T, tolerant; D, deleterious; P, pathogenic; DM, disease-causing mutation. ^a^ Pathogenicity prediction for missense variants based on in silico algorithms, METALR and METASVM, which are ensemble prediction scores that incorporate results from nine algorithms and allele frequency. CADD was also applied. ^b,c^ Genome Aggregation Database (gnomAD) exomes and genomes, respectively, in East Asian population. Structural interaction: variants that impact the internal interactions of the resulting polypeptide structure.

## Data Availability

According to US NIH policy, the exome sequencing data for the European ancestry dataset will be released to the database of Genotypes and Phenotypes (dbGAP).

## Supplementary Materials

The following are available online at https://www.mdpi.com/article/10.3390/cancers13112704/s1, Supplementary Table S1: Details of the criteria for classification of pathogenicity categories, Supplementary Table S2: Genes evaluated in this study and their biological functions, Supplementary Table S3: Details of variants identified in the European ancestry dataset, Supplementary Table S4: Details of potentially pathogenic variants in the Chinese dataset, Supplementary Table S5: Top genes with higher burden of variants.
